# Aberrant gene expression profiles, during in vitro osteoblast differentiation, of telomerase deficient mouse bone marrow stromal stem cells (mBMSCs)

**DOI:** 10.1186/s12929-015-0116-4

**Published:** 2015-01-30

**Authors:** Hamid Saeed, Mehwish Iqtedar

**Affiliations:** Endocrine Research Laboratory, KMEB, Department of Endocrinology and Metabolism, Odense University Hospital, Odense, Denmark; University College of Pharmacy, Punjab University, Allama Iqbal Campus, 54000 Lahore, Pakistan; Department of Bio-technology & Microbiology, Lahore College for Women University, Lahore, Pakistan

**Keywords:** Telomerase, Telomeres, BMSCs, Mesenchymal stem cells, Aging, Osteoblast

## Abstract

**Background:**

Telomerase deficiency has been associated with inadequate differentiation of mesenchymal stem cells. However, the effect of telomerase deficiency on differential regulation of osteoblast specific genes, based on functional gene grouping, during *in vitro* osteoblast differentiation has not been reported before.

**Results:**

To examine these effects, *Terc*^*-/-*^ BMSCs (bone marrow stromal stem cells) were employed which exhibited reduced proliferation during *in vitro* osteogenesis along with increased population doubling time and level compared to wild type (WT) BMSCs during the normal culture. Osteogenic super array at day 10 of osteoblast differentiation revealed that telomerase deficiency strongly affected the osteoblast commitment by down-regulating *Runx2, Twist* and *Vdr* – known transcription regulators of osteogenesis. Similarly, in *Terc*^*-/-*^ BMSCs a marked reduction in other genes engaged in various phases of osteoblast differentiation were observed, such as *Fgfr2* involved in bone mineralization, *Phex* and *Dmp1* engaged in ossification, and *Col11a1* and *Col2a1* involved in cartilage condensation. A similar trend was observed for genes involved in osteoblast proliferation (*Tgfb1, Fgfr2* and *Pdgfa*) and bone mineral metabolism (*Col1a1, Col2a1, Col1a2* and *Col11a1)*. More profound changes were observed in genes engaged in extracellular matrix production: *Col1a1, Col1a2, Mmp10, Serpinh1* and *Col4a1*.

**Conclusion:**

Taken together, these data suggest that telomerase deficiency causes impairment of BMSCs differentiation into osteoblasts affecting commitment, proliferation, matrix mineralization and maturation. Thus, modulating telomerase in BMSCs with advanced aging could improve BMSCs responsiveness towards osteoblast differentiation signals, optimal for osteoblast commitment, proliferation and maturation processes.

**Electronic supplementary material:**

The online version of this article (doi:10.1186/s12929-015-0116-4) contains supplementary material, which is available to authorized users.

## Background

Osteoporosis is a disease of increased bone/skeletal fragility common in the elderly and estrogen deficient postmenopausal women [1,2]. Bone homeostasis is strictly maintained by a well coordinated venture and steadiness between bone formation by osteoblasts and bone resorption by osteoclasts [3]. However, disruption in any of these above mentioned processes leads to skeletal/bone fragility i-e., osteoporosis [3]. In this regard, role of osteoclasts in bone resoption have extensively been studied and many treatments are targeted at preventing bone resorption, while little attention is paid towards bone anabolic effects by enhancing osteoblasts and osteocytes functions [3,4]. Therefore, equally important is to study the physical factors and instigators that can contribute towards a decline in osteoblast functions with advanced aging. Osteoblasts are derived from bone marrow stromal cells (BMSCs) also known as mesenchymal stem cells (MSCs) capable of differentiating into multiple lineages i-e., osteoblasts, chondrocytes and adipocytes [5], thus acting as the primary source of skeletal repair [6].

Tissue homeostasis is exceptionally maintained by a strict balance between cell loss and cell replacement during the course of tissue/organ life [7,8]. However, with aging and in degenerative diseases, this balance declines progressively resulting in reduced supply of new cells to compensate the lost/dead cells, resulting in impaired tissue integrity and function along with reduced regeneration capacity upon damage [9]. Therefore, with aging, owing to several factors including telomerase deficiency, functional and numerical decline of BMSCs resulted in the compromised ability of BMSCs to repair the skeleton and maintain skeletal homeostasis [10-12].

Telomerase was first discovered by Greider et al. [13] in the extracts of the protozoan *Tetrahymena thermophila*. Telomerase plays an essential role in the maintenance of choromosomal ends that is telomeres - G-rich simple repeat sequences (TTAGGG) that are synthesized by a special reverse transcriptase called *Telomerase* [14,15]. This enzyme requires a template to act which is the RNA component of telomerase i.e, *TERC* [15,16]. Telomerase is inactive in most somatic cells but active in germ cells, stem cells and actively dividing cells [17]. Telomerase deficient mice (*Terc-/-*) have been instrumental in delineating the impact of telomere shortening in context of whole organism [18]. Disease states that appear in *Terc*^*-/-*^ reiterate the disease states, with more or less same etiology, in humans characterized by short telomere in the cells as a result of excessive proliferation [19-21].

For clinical purposes, BSMCs are expanded *in vitro* to attain the desired number of cells which are not available from young marrow donors or through any other source, whereas the frequency and quality of BMSCs decline further with aging [22,23]. Yet, little attention is paid towards *in vitro* differentiation hitches encountered by BMSCs during the course of aging with telomerase deficiency. In this study, we performed osteogenic transcriptional profiling of telomerase deficient BMSCs during *in vitro* osteoblast differentiation and found significant disruption in normal osteogenic gene expression profiles. Data suggest that telomerase deficiency in BMSCs can cause noteworthy effect on the expression of genes vital for skeletal development, bone mineral metabolism, cell growth and differentiation, extra-cellular matrix production and transcription regulation to initiate and maintain normal osteogenesis.

## Methods

### Mice breeding, genotyping & handling

*Terc* deficient mice (*Terc*^*-/-*^ Strain-004132) were purchased from Jackson laboratory and kept in a pathogen-free environment on standard chow. *Terc*^*-/-*^ were inter-crossed to generate 3^rd^ generation *Terc*^*-/-*^ (*Terc*^*-/-*^*-* G3) mice, and were maintained in a C57BL/6J background. Wild type *Terc*^*+/+*^ mice were employed as controls. Genotyping was performed according to the protocol recommended by Jackson laboratory. NOD/MrkBomTac-*Prkdc*^*scid*^ mice (NOD/SCID mice) were purchased from Taconic, Denmark. The Danish Animal ethical committee approved all the mouse experiments.

### Bone marrow stromal cell isolation

Bone marrow stromal cells were harvested according to the protocol described by Peister [24], with some in house modifications [25]. Media was changed for every 3^rd^ day, subsequently after 1 to 2 weeks cells were dissociated using Trypsin/EDTA for 4 min at 37°C and plated according to the experimental setup.

### Osteoblast differentiation

Osteogenic differentiation of BMSCs; cells were plated at high densities; 20 × 10^3^ cells/cm^2^ in 24 well plates for staining and in 6 well plates for RNA harvesting containing complete expansion media (CEM): Iscove modified Dulbecco medium (IMDM; GIBCO, Cat. No. 21980) containing 12% FBS (FBS; GIBCO), 100 U/ml penicillin (GIBCO), 100 μg/ml streptomycin (GIBCO) and 12 μM L-glutamine (GIBCO, Cat. No. 25030) supplemented with osteogenic cocktail; 10 nM dexamethasone (Sigma), 10 mM β – glycerol-phosphate (Sigma) & 50 μg/ml Vitamin C (Sigma). Media was changed every 3^rd^ day until day 10 and then cells were stained for Alkaline phosphatase (ALP) and harvested for RNA isolation.

### Cytochemical staining

#### Alkaline phosphatase staining

Cells were fixed with acetone/citrate buffer pH 4.2 (11/2:1) for 5 min at room temperature and stained with Naphtol-AS-TR-phosphate solution for 1 h at room temperature. Naphtol-AS-TR-phosphate solution consists of Naphtol-AS-TR-phosphate (Sigma) diluted 1:5 in H2O and Fast Red TR (Sigma) diluted 1:1.2 in 0.1 M Tris buffer (OUH pharmacy), pH 9.0, where both solutions were mixed 1:1. Cells were counterstained with Mayers-Hematoxylin for 5 min at room temperature.

### Alizarin red staining for mineralized matrix

Cells were fixed with 70% ice-cold ethanol for 1 h at −20°C, and stained with 40 mM alizarin red S (AR-S; Sigma), pH 4.2 for 10 min at room temperature.

### Quantitative real time mouse osteogenesis RT^2^ profiler™ PCR array

Quantitative mRNA expression of 84 genes related to osteogenic differentiation was performed using Mouse osteogenesis RT^2^ profiler™ PCR array (Super array Bioscience Corporation, Frederick, MD, USA) [26,27]. RNA was isolated from WT and *Terc*^*-/-*^ BMSCs (*n* = 3), at day 10 of osteoblast differentiation, using Trizol® and according to the manufacture’s protocol. Concentration of RNA was determined spectrophometrically by absorbance at 260 nm with GeneQuant pro (Biochrom Ltd.). First strand complementary DNA was synthesized from 2 μg of total RNA using a commercial revertAid H minus first strand cDNA synthesis kit (Fermentas, Helsingborg, Sweden), according to the manual’s instructions. cDNA from three biological replicates were pooled and PCR array analysis was done according to manufacturer instructions with RT^2^ Real Time™ SYBER GREEN PCR Master mix (Super array Biosciences, Corporation). Quantitative Real Time PCR array was performed in an iCycler IQ detection system (Bio-Rad, Herlev, Denmark).

## Results

### Defective proliferation of *Terc*^*-/-*^ BMSCs during *in vitro* osteoblast differentiation

**Figure 1:**
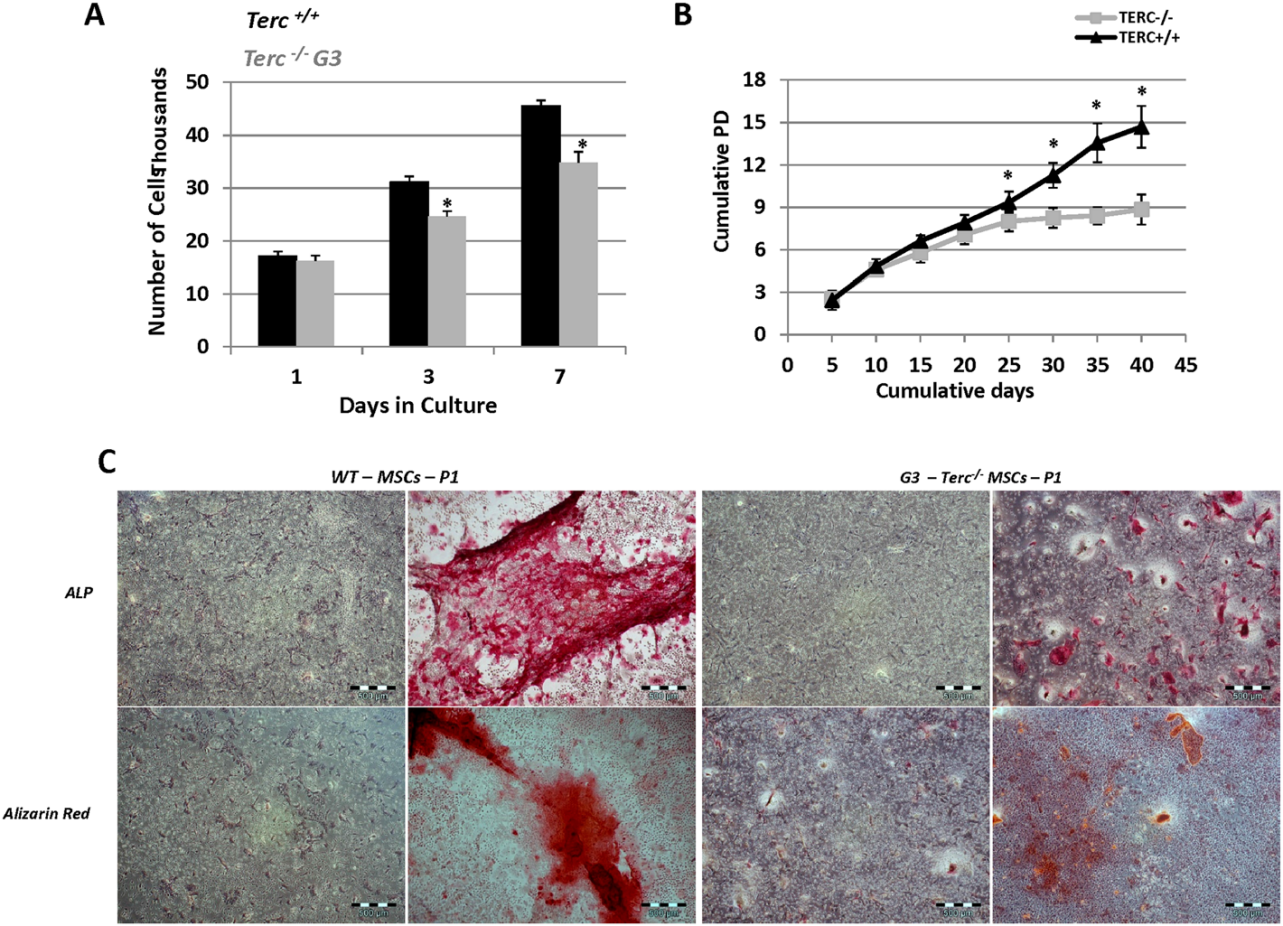
**Telomerase deficient BMSCs exhibited impaired** ***in vitro*** **osteoblast differentiation and proliferation with reduced population doubling time and level. A)** Short term proliferation of *Terc* ^*-/*^ and WT BMSCs, equal number of cells were plated at day 0 and counted at day 1, 3 and 7. **B)** Long term proliferation of *Terc* ^*-/-*^ and WT BMSCs. Population doubling time (PDT); *Terc* ^*-/-*^ BMSCs PDT of 2.1 days compared to PDT of 1.89 days by WT BMSCs at early passage and later increased to 23 and 3.8 days, respectively. **C)** *In vitro* osteoblast differentiation of *Terc* ^*-/-*^ and WT BMSCs - according to the protocol described in material and methods. Alkaline phosphatase (ALP) (upper row) and Alizarin Red (lower row) staining at day 10 of osteoblast differentiation. Data are represented of means ± SD of three independent experiments. *p ≤ 0.05.

We have previously shown that *Terc*^*-/-*^ BMSCs exhibit stunted proliferation as evident by BrdU labelling during normal culture [12], while proliferation was not assessed during *in vitro* osteoblast differentiation. To examine the proliferation rate during *in vitro* osteogenesis, we differentiated WT and *Terc*^*-/-*^ BMSCs into osteoblasts and counted cells at different time points. *Terc*^*-/-*^ BMSCs demonstrated reduced proliferation rate compared to WT controls at day 3 and day 7 (Figure 1A). Moreover, population doubling analysis during normal culture revealed that *Terc*^*-/-*^ BMSCs took more time to double with population doubling level (PDL) of 2.44 initially to PDL of 0.214 after 40 days compared to WT BMSC’s PDL of 2.64, which later dropped to PDL of 1.3 after 40 days (Figure 1B). Similarly, population doubling time (PDT) of *Terc*^*-/-*^ BMSCs was 2.1 days, and later increased to 23 days at later passages, while PDT of WT BMSCs was around 1.89 days at early passage and 3.8 days around day 40 (Figure 1B). Also cumulative population doubling (CPD) stands at 8.62 for *Terc*^*-/-*^ BMSCs at day 40 compared to 15.4 for WT BMSCs at day 40 (Figure 1B).

### Telomerase deficiency causes atypical osteoblast differentiation of BMSCs

We have previously shown that telomerase deficient BMSCs and bones exhibit signs of defective osteogenesis with more detailed data on long bones compared to BMSCs [12]. Thus we aimed at delving into more detailed study of telomerase deficient BMSCs. Next task was to select a time point that could detect most of the super array genes to conclude, connect and delineate the functional dysregulation of genetic molecules during osteoblast differentiation due to telomerase deficiency. Thus we did a pilot experiment on WT BMSCs differentiation into osteoblasts considering three different time points (Additional file 1: Figure S1A); day 3, 7 and 10, and selected day 10 to perform super array based on observed data. We differentiated *Terc*^*-/-*^ BMSCs into osteoblasts until day 10, where we analyzed ALP activity by ALP staining and matrix mineralization by Alizarin Red staining. *Terc*^*-/-*^ BMSCs showed decreased *in vitro* osteoblast differentiation compared to WT BMSCs at day 10 (Figure 1C).

### Osteogenic super array gene profiling displayed defective osteoblast differentiation and function in *Terc*^*-/-*^ BMSCs

**Figure 2:**
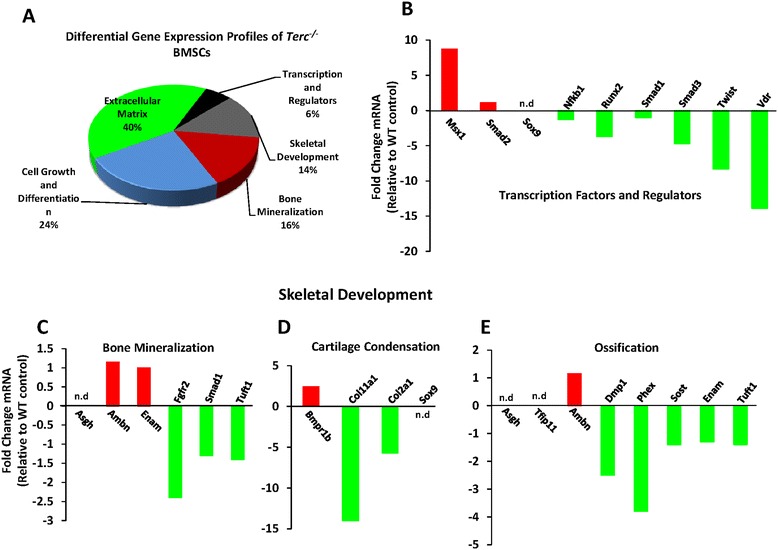
**Differential gene expression profiles of telomerase deficient BMSCs employing mouse osteogenesis RT** ^**2**^ **profiler™ PCR array. A)** Pei chart showing differential gene expression profiles associated with each stage of osteogenesis. **B)** Differentiation gene expression profiles of transcription factors and regulators in *Terc* ^*-/-*^ BMSCs up-regulated (red bars) and down-regulated (green bars) relative to WT controls. **C-E)** Skeletal development genes, known to be involved in bone mineralization, cartilage condensation and ossification, up-regulated (red bars) and down-regulated (green bars) in *Terc* ^*-/-*^ BMSCs relative to WT controls. Genes that were not amplified or not detectable in the PCR array were marked as *‘n.d’* (not detectable). Osteogenic super array data are represented as fold change relative to WT controls of three independent biological replicates pooled together. Panel **(A)** C = control, I = induced, scale bar 500 μm.

**Figure 3:**
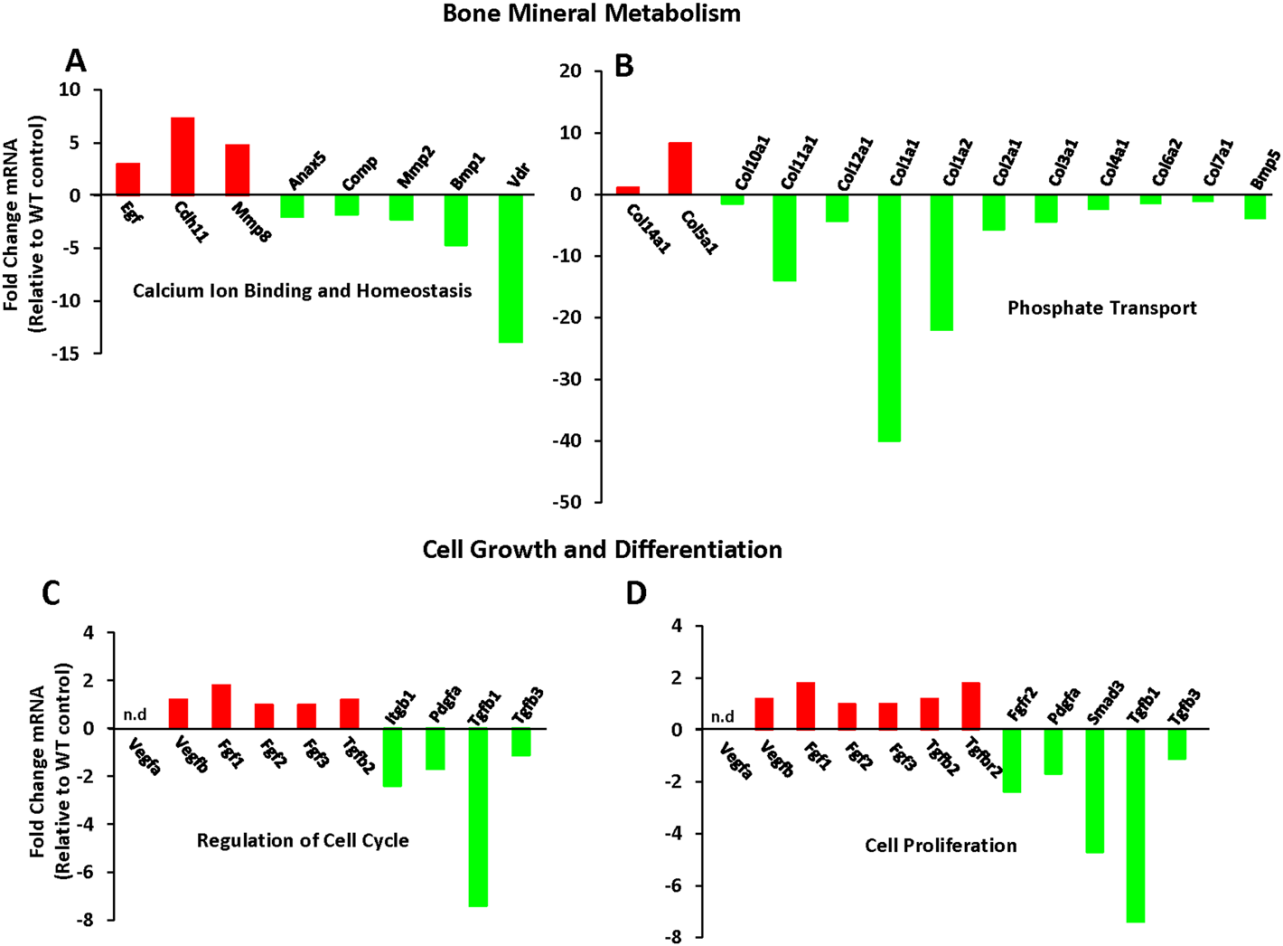
**Differential expression of genetic molecules involved in bone mineral metabolism and cell growth during differentiation in** ***Terc*** ^***-/-***^ **BMSCs. A & B)** Differential expression of genes involved in bone mineral metabolism affecting calcium ion binding and phosphate transport in *Terc* ^*-/-*^ BMSCs compared to WT controls. **C-D)** Differential transcriptional profiles of *Terc* ^*-/-*^ BMSCs during *in vitro* osteoblast differentiation. Several genes with known involvement in osteoblast cell cycle, proliferation and differentiation were up-regulated (red bars) and down-regulated (green bars) in *Terc* ^*-/-*^ BMSCs compared to control. Genes that were not amplified or not detectable in the PCR array were marked as *‘n.d’* (not detectable). Osteogenic super array data are represented as fold change relative to WT controls of three independent biological replicates pooled together.

**Figure 4:**
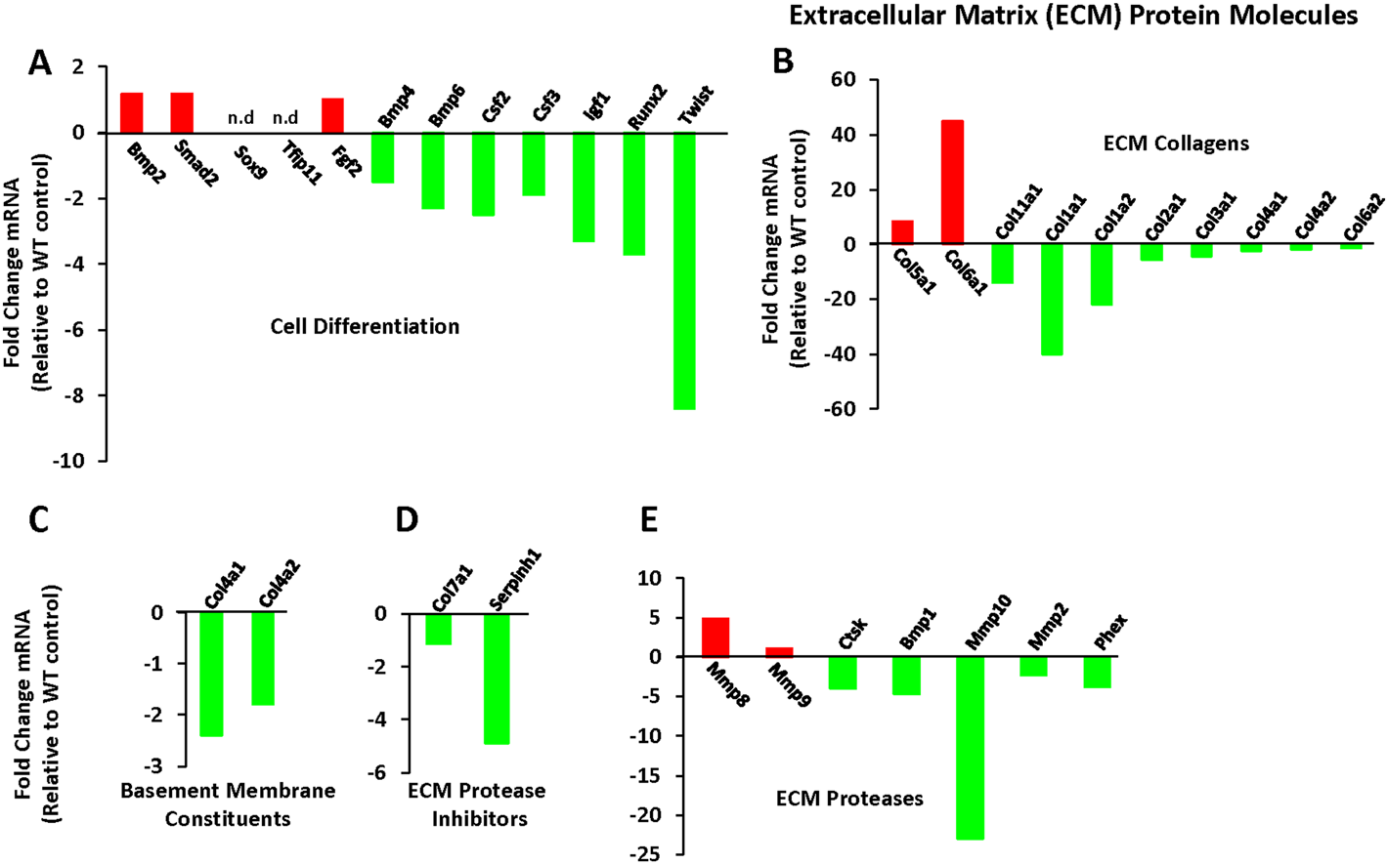
**Effect of telomerase deficiency on extracellular matrix (ECM) protein molecules during** ***in vitro*** **osteoblast differentiation of BMSCs. A)** Differential expression of genes involved in cell differentiation into ostoeblasts. **B-E)** Differential gene expression profiles of molecules involved in extracellular matrix production in *Terc* ^*-/-*^ BMSCs – notable genetic molecules were related with ECM collagens, basement membrane constituents, ECM proteases, ECM protease inhibitors and other molecules associated with ECM. Genes that were not amplified or not detectable in the PCR array were marked as *‘n.d’* (not detectable). Osteogenic super array data are represented as fold change relative to WT controls of three independent biological replicates pooled together.

**Figure 5:**
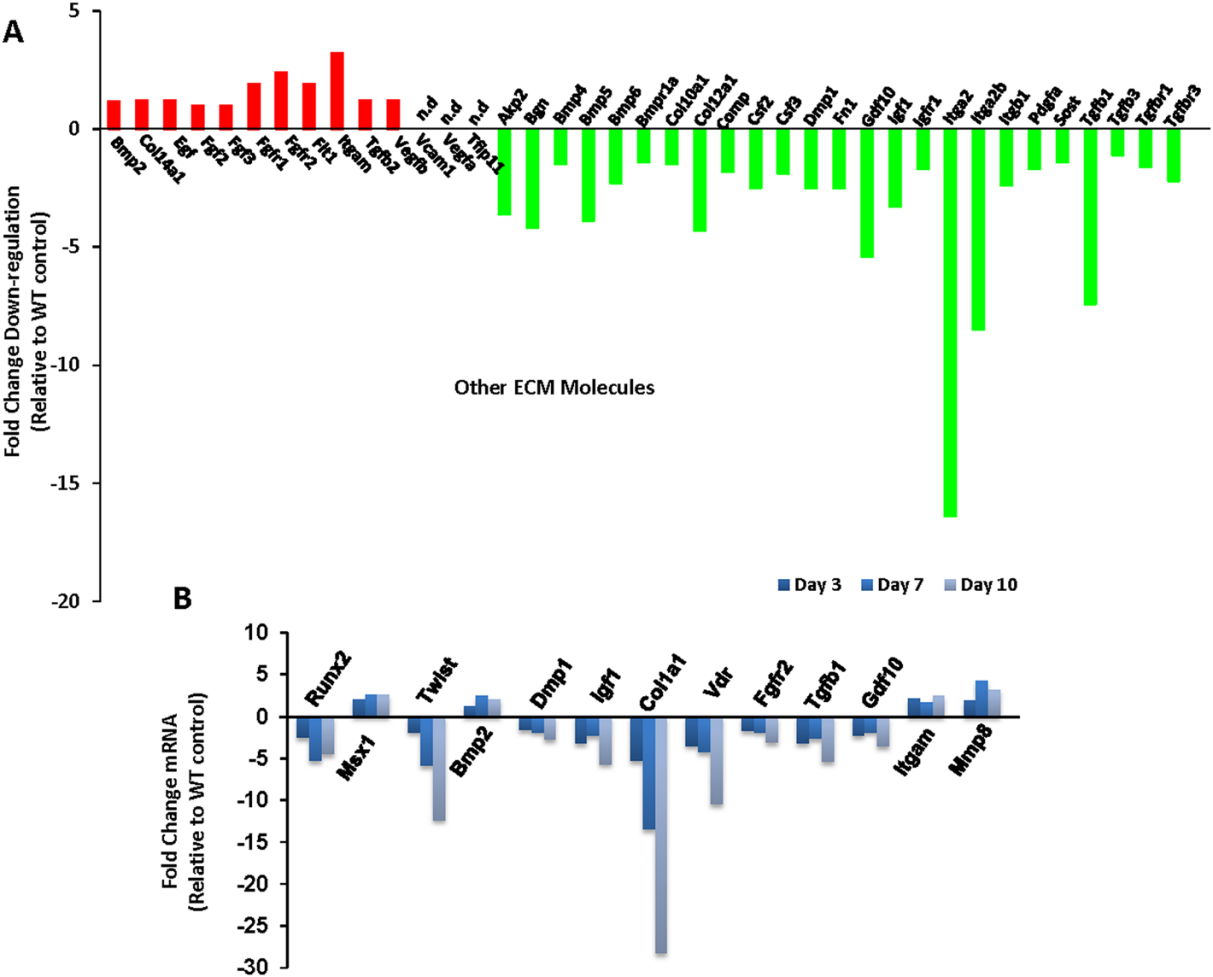
**Other extracellular matrix (ECM) protein molecules affected by telomerase deficiency and differentiation time point re-ascertainment by real time PCR. A)** Other ECM molecules affected by Telomerase deficiency. **B)** Re-ascertainment of *in vitro* differentiation time point by comparing three time points Day 3, 7 and 10 employing both control and *Terc* ^*-/-*^ BMSCs. Genes that were not amplified or not detectable in the PCR array were marked as *‘n.d’* (not detectable). Osteogenic super array data are represented as fold change relative to WT controls of three independent biological replicates pooled together.

To study further, the effects of telomerase deficiency during *in vitro* osteoblast differentiation we performed Mouse Osteogenesis RT^2^ Profiler™ PCR Array as described in material and methods. Data analysis revealed defective osteoblast differentiation with differential expression of several genes at various phases of osteoblast differentiation. Briefly, major changes were observed in genes controlling extracellular matrix production which accounted for 40% of the genes, followed by cell growth and differentiation (24%), bone mineralization (16%), skeletal development (14%) and transcription factors and regulators (6%) respectively (Figure 2A). Moreover, during PCR super array several of the genes were not amplified or detectable and were marked as ‘n.d’ (not detectable) in the figures (Figures 2, 3, 4 and 5). Moreover, results are arranged and described according to functional gene grouping specified by super array bioscience cooperation.

#### Transcription factors and regulators

Noteworthy genes that affected this category include, runt related transcription factor 2 (*Runx2, -3.7*), smad family member 3 (*Smad3, -4.7*), twist – basic helix loop helix transcription factor (*Twist, -8.3*) and vitamin D receptor (*Vdr, -13.9*) (Figure 2B).

#### Skeletal development

Several genes were found to be differentially expressed - known to be involved in bone mineralization, cartilage condensation and ossification (Figure 2C-E). Notable genes that were down-regulated in *Terc*^*-/-*^ BMSCs include fibroblast growth factor receptor 2 (*Fgfr2, -2.4*) important for bone mineralization, collagen type 11 alpha 1 (*Col11a1, -14*), collagen type 2 alpha 1 (*Col2a1, -5.7*), dentine matrix protein (*Dmp1, -2.5*) and phosphate regulating neutral endopeptidase on chromosome X (*Phex, -3.8*) involved in cartilage condensation (Figure 2C-E). Other noteworthy genes include runt related transcription factor 2 (*Runx2, -3.7*), bone morphogenetic protein 2 (*Bmp4, -1.5*), bone morphogenetic protein 6 (*Bmp6, -2.3*), transforming growth factor beta 1 (*Tgfb1, -7.4*) and vitamin D receptor (*Vdr. -13.9*) (Additional file 1: Figure S1B).

#### Bone mineral metabolism

Similarly, expression level of several genes known to be involved in calcium ion binding and phosphate transport were reduced in *Terc*^*-/-*^ BMSCs compared to WT (Figure 3A & B). Most significantly affected (down-regulated) genes in calcium ion binding category include cartilage oligomatrix protein (*Comp, -1.8*), matrix metallopeptidase 2 (*Mmp2, -2.3*), bone morphogenetic protein 1 (*Bmp1, -4.7*) and vitamin D receptor (*Vdr, -13.9*). In phosphate transport category collagens were adversely affected – notable down-regulated genes include collagen type 1 alpha 1 (*Col1a1, -40*), collagen type 1 alpha 2 (*Col2a1, -22*), collagen type 2 alpha 1 (*Col2a1, -5.7*) and bone morphogenetic protein 5 (*Bmp5, -3.9*) (Figure 3A & B).

#### Cell growth and differentiation

Genes that were involved in cell cycle regulation and were down-regulated in *Terc*^*-/-*^ BMSCs include integrin beta 1 (*Itgb1,* -*2.4*), transforming growth factor beta 1 (*Tgfb1, -7.4*) and platelet derived growth factor alpha (*Pdgfa, -1.7*) (Figure 3C & D). Several growth factors and receptors engaged in cell proliferation and differentiation were also down-regulated in *Terc*^*-/-*^ BMSCs, such as colony stimulating factor 2 (*Csf2, -2.5*), colony stimulating factor 3 (*Csf3, -1.9*), bone morphogenetic protein 4 (*Bmp4, -1.5*), bone morphogenetic protein 6 (*Bmp6, -2.3*), insulin like-growth factor 1 (*Igf1, -3.3*), insulin like-growth factor receptor 1 (*Igfr1, -1.7*), transforming growth factor beta receptor 3 (*Tgfbr3, -2.17*) and growth differentiation factor 10, (*Gdf10, -5.4*) (Figure 4A and Additional file 1: Figure S1C). Similarly, genes important for cell differentiation were also markedly reduced in *Terc*^*-/-*^ BMSCs compared to WT BMSCs, most conspicuous were bone morphogenetic protein 4 (*Bmp4, -1.5*), bone morphogenetic protein 6 (*Bmp6,- 2.3*), Insulin like-growth factor 1 (*Igf1, -3.3*), runt related transcription factor 2 (*Runx2, -3.7*) and twist – basic helix loop helix transcription factor (*Twist, -8.4*) (Figure 4A and Additional file 1: Figure S1C).

#### Extracellular matrix (ECM) protein

Among the ECM proteins, collagens were most significantly affected in *Terc*^*-/-*^ BMSCs compared to WT BMSCs (Figures 4B-E & 5A). Most significant reduction was observed in collagen type 11 alpha 1 (*Col11a1, -14*), collagen type 1 alpha 1 (*Col1a1, -40*), collagen type 1 alpha 2 (*Col1a2, -22*) and collagen type 2 alpha 1 (*Col2a1, -5.7*). Among ECM proteases, matrix metallopeptidase 10 (*Mmp10, -23*) was markedly reduced in *Terc*^*-/-*^ BMSCs (Figures 4B-E & 5A). Other most conspicuous molecules crucial for ECM production include, biglycan (*Bgn, -4.2*), alkaline phosphatase (*Akp2, -3.6*), cartilage oligomatrix protein (*Comp, -1.8*), dentine matrix protein 1 (*Dmp1, -2.5*), integrin alpha 2 (*Itga2, -16.4*), integrin alpha 2b (*Itga2b, -8.5*), collagen type 12 alpha 1 (*Col12a1, -4.3*) (Figures 4B-E & 5A).

### Re-ascertainment of data analysis time point by real time PCR

We already compared genes expression at three different time points (day 3, 7 and 10) employing control samples, where suitable expression level can be ascertained to perform data analysis (Additional file 1: Figure S1A). However, next obvious question was to re-confirm the appropriateness of data analysis time point, using control and test samples, by comparing different time points; day 3, day 7 and day 10. Therefore, we selected two genes from each functional gene grouping category of osteogenic super array for comparative gene expression analysis using Real time PCR. Real time PCR data further suggest, that under available resources and time point options, day 10 provide better reckoning of osteoblast specific expression of genes (*Runx2, Msx1, Bmp2, Dmp1, Igf1, Col1a1, Vdr, Gdf10 and Mmp8*) pertinent for investigating the role of telomerase deficiency during *in vitro* osteoblast differentiation (Figure 5B).

## Discussion

Telomerase deficiency has been shown to effect BMSCs differentiation into osteoblast, adipocytes and chondrocytes [28]. We have previously shown that telomerase deficiency causes reduced proliferation, enhanced senescence and up-regulation of cell cycle inhibitors in telomerase deficient BMSCs [12], while its over-expression has been shown to enhance BMSCs proliferation and osteogenic differentiation; both *in vitro* and *in vivo*, with no signs of *in vitro* senescence [29]. In this study, we performed more detailed expression profiling of dysregulated genes affected during *in vitro* osteoblast differentiation in *Terc*^*-/-*^ BMSCs. Data suggested that telomerase deficiency caused more significant dysregulation in genes involved in osteogenic commitment and extracellular matrix production during the differentiation process.

Osteoblastogenesis is a multifaceted and intricate process regulated by temporal and spatial expression of transcription factors, cytokines, growth factors, hormones and morphogens in a stage specific manner [30,31]. Subtle differences in any of these factors can affect the coordinated effort towards lineage commitment to lineage maturation. Our data demonstrated that telomerase deficiency caused differential gene expression profiles emanating from dysregulation of transcription regulation to extracellular matrix production and bone mineralization during the course of *in vitro* osteoblast differentiation. Reduction in *Runx2, Twist* and *Vdr* in *Terc*^*-/-*^ BMSCs, possibly suggest initial inadequacy in osteogenic lineage commitment. Nevertheless, *Msx1*, another transcription regulator, was significantly up-regulated in *Terc*^*-/-*^ BMSCs with documented role in cell proliferation and differentiation during embryonic development, affecting *Runx2* expression in neural crest cells and during osteoblast differentiation [32]. Thus, up-regulation of *Msx1* in *Terc*^*-/-*^ BMSCs could suggest a positive feedback signal to enhance *Runx2* expression, since *Runx2* lies downstream of *Msx1* [33]. Additionally, down-regulation of *Twist* in *Terc*^*-/-*^ BMSCs presumably favour osteoblast specific genes expression because literature evidence suggests that down-regulation of *Twist* genes (*Twist1* and *Twist2*) is required to initiate osteoblast specific gene expression [34]. These data suggest that telomerase deficiency causes inadequate transcriptional control over BMSCs lineage commitment towards osteoblast. However, insensitivity of mesenchymal progenitors towards the differentiation signals could possibly come from the senescent cells. It is plausible that senescent cells secrete several inhibitory cytokines and mortifying enzymes affecting cell responses towards the inducing signals - ending up in the reduction of agile progenitor’s pool that could respond to give a more affirmative response towards the induction signals.

Furthermore, marked reduction in genes involved in cellular growth, differentiation and skeletal development such as, *Pdgfa, Itgb1, Smad3*, *Dmp1, Phex, Fgfr2, Col11a1* and *Tgfb1*, suggest that telomerase deficiency resulted in defects from proliferative phase to mineralization phase of osteogenesis. For example, *Itgb1* has been shown to promote and regulate cell spreading, proliferation and cytoskeleton integrity to influence cell differentiation [35], *Dmp1* is vital for mineralization of bone and later functioning of osteocytes crucial for proper bone re-modelling process [36]. The reduced levels of *Fgfr2* mRNA, upon deletion of *Twist*, have been associated with decreased mRNA levels of *Runx2, Bsp* and *Oc* [37]. *Col11a1* has been shown to suppress terminal osteoblast differentiation which is activated by *Lef1* via direct physical interaction of *Lef1* with *Col11a1* promoter sans b-catenin [38]. Seemingly, there exist numbers of genetic signals, in *Terc*^*-/-*^ BMSCs, opposing or affecting osteoblast differentiation processes. While in parallel there were signals with insisting support for osteoblast differentiation process as evident from various gene expression profiles, such as down-regulation of *Col11a1,* which suppresses terminal osteoblast differentiation [38]*,* down-regulation of *Tgfb1*, known to muffle osteoblastic proteins, especially *Runx2* via *Smad3* [39] to further suppress *Alp* and *Oc* expression [38,40] and up-regulation of *Egf, Fgfr2* and *Fgf1*, known pro-mitogenic signals [41-43], vital for osteoprogenitors expansion to attain osteoblast maturation stage. Furthermore, these pro-osteoblastic efforts were reinforced by up-regulation of other genes such as *Bmpr1b, Col5a1* and *Cdh11* known to support osteoblast differentiation process at various phases and by distinct mechanisms [44-46]. Besides, these data should be interpreted with great care owing to cell culture heterogeneity and variation in telomere lengths that can result in muddled responses. Nevertheless, osteoblast suppressive responses are dominant over signals favouring osteoblast differentiation process. Moreover, it would be interesting to have different cell culture options with differences in telomerase inflection to better understand the dynamics of telomerase enzyme and it’s physiologically relevant enzymatic activity that could possibly favour optimal osteoblast differentiation.

Furthermore, extracellular matrix is a central component of cellular microenvironment that plays crucial role in cell behaviour and function by regulating cell adhesion, migration, apoptosis, proliferation and differentiation [47]. Strikingly, several collagens associated with ECM, such as *Col1a1, Col1a2, Col2a1* and *Col11a1* were dysregulated in our data. Among all the collagens present in the ECM, Collagen type 1 (*Col1a1*) is a major protein present in the ECM of the bone and plays an important role in bone mineralization [48]. Similarly, *Itga2* deficiency in mice, another component of ECM - significantly reduced in *Terc*^*-/-*^ BMSCs, has been shown to reduce joint pathology, such as reduction in pannus formation, joint inflammation and cartilage erosion [49]. Our data and literature evidences suggest that telomerase deficiency causes severe modifications in ECM related collagens and integrins that could also synergise the unfavourable outcome regarding BMSCs proliferation, survival and differentiation, since ECM has much demanding role in osteoblast differentiation rather than any other cell differentiation process. Moreover, data also suggested the presence of mixed signals, ECM protective and EMC un-protective. For example, down-regulation of *Serpinh1*, an ECM protease inhibitor involved in ECM degradation, implicated in osteogenesis imperfecta and encodes collagen chaperone protein *Hsp47* [50] and up-regulation of *Mmp8* - alleviating joint inflammation and bone erosion [51] coupled with down-regulation of *Mmp10* involved in promoting cartilage degradation [52], respectively.

## Conclusion

In conclusion, telomerase deficiency causes inadequate *in vitro* differentiation of BMSCs into osteoblasts manifested by dysregulation of genes involved in various phases of osteoblast differentiation. It is plausible that initial defect at transcriptional level ramify into regulatory changes in the expression of genes associated with specific phases of the differentiation process. It is also likely, that telomerase deficient cells undergoing senescence are not actively participating in the differentiation process, thus lacking optimal signal threshold levels suitable for osteoblast commitment and proliferation, thereby affecting osteoprogenitors pool size. Data also suggest that *Terc*^*-/-*^ BMSCs seems quite perplexed in their response towards differentiating signals because osteoblast suppressive and osteoblast promoting signals were evident from the super array PCR data or dual responses suggest a positive and negative feedback loop mechanisms to assimilate a coordinated effort towards osteoblast differentiation. However, it is possible that *Terc*^*-/-*^ BMSCs in culture exhibit differences in their telomere lengths, where cells having critically short telomeres undergoing senescence are associated with gene regulation oblivious of osteoblast differentiation, while cells having optimal lengths are giving due responses towards the differentiation signals. Other likely possibility is the conformational changes in the chromatin owing to telomere shortening, thus regulating undesirable gene expression pattern altering normal response of BMSCs towards osteogenic differentiation. Therefore, regulating telomerase expression at optimal physiological levels to attain uniform telomere lengths, using molecular, chemical and pharmacological ways, and to avoid senescence and malignant transformations could further improve our understanding of the mechanisms associated with defective differentiation due to telomerase deficiency and telomere length dynamics.

## Additional file

Additional file 1: Figure S1. Telomerase deficiency in BMSCs caused defects in skeletal development genes and several growth factors and receptor molecules involved in cellular growth and differentiation. **A)** Gene expression profiles of WT BMSCs at three different time points during *in vitro* Osteoblast differentiation. **B)** Genes affected by telomerase deficiency in BMSCs during their *in vitro* osteogenesis involved in skeletal development. **C)** Notable growth factors and receptor molecules affected in *Terc* ^*-/-*^ BMSCs and having association with cellular growth and differentiation process. Genes that were not am,plofied or not detectable in the PCR array were marked as *‘n.d’* (not detectable). Osteogenic super array data are represented as fold down-regulation relative to WT controls of three independent biological replicates pooled together.
