# Supplementary material for: Aberrant gene expression profiles, during in vitro osteoblast differentiation, of telomerase deficient mouse bone marrow stromal stem cells (mBMSCs)
Source: J Biomed Sci. 2015 Jan 30;22(1):11. doi: 10.1186/s12929-015-0116-4 (PMC4318164; doi:10.1186/s12929-015-0116-4)
Supplement: Additional file 1: Figure S1. — Telomerase deficiency in BMSCs caused defects in skeletal development genes and several growth factors and receptor molecules involved in cellular growth and differentiation. A) Gene expression profiles of WT BMSCs at three different time points during in vitro Osteoblast differentiation. B) Genes affected by telomerase deficiency in BMSCs during their in vitro osteogenesis involved in skeletal development. C) Notable growth factors and receptor molecules affected in Terc -/- BMSCs and having association with cellular growth and differentiation process. Genes that were not am,plofied or not detectable in the PCR array were marked as ‘n.d’ (not detectable). Osteogenic super array data are represented as fold down-regulation relative to WT controls of three independent biological replicates pooled together. [file 12929_2015_116_MOESM1_ESM.doc]

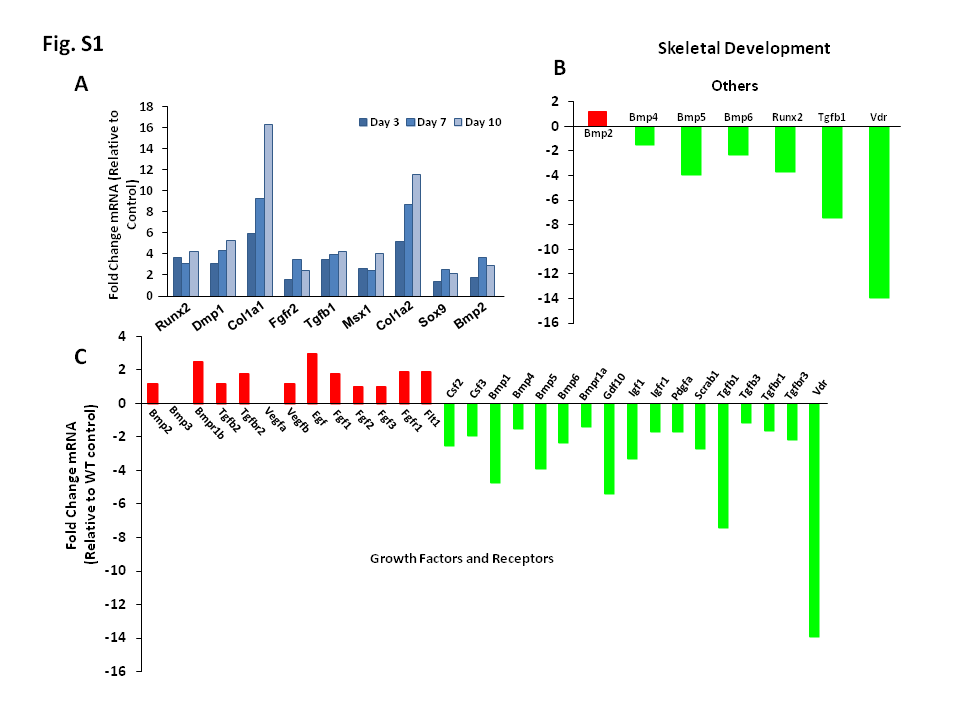


**Figure S1. Telomerase deficiency in BMSCs caused defects in skeletal development genes and several growth factors and receptor molecules involved in cellular growth and differentiation. A)** Gene expression profiles of WT BMSCs at three different time points during in vitro Osteoblast differentiation**. B)** Genes affected by telomerase deficiency in BMSCs during their *in vitro* osteogenesis involved in skeletal development. **C)** Notable growth factors and receptor molecules affected in *Terc^-/-^* BMSCs and having association with cellular growth and differentiation process. Genes that were not am,plofied or not detectable in the PCR array were marked as *‘n.d’* (not detectable). Osteogenic super array data are represented as fold down-regulation relative to WT controls of three independent biological replicates pooled together.
